# Activity of Natural Substances and n-Undecyl-α/β-l-Fucopyranoside Against the Formation of Pathogenic Biofilms by *Pseudomonas aeruginosa*

**DOI:** 10.3390/antibiotics15010076

**Published:** 2026-01-10

**Authors:** Christian Dietrich Vogel, Anne Christine Aust, Raffael Christoph Wende, Undraga Schagdarsurengin, Florian Wagenlehner

**Affiliations:** 1 Department of Urology, Pediatric Urology and Andrology, Justus Liebig University, Rudolf-Buchheim-Strasse 7, 35392 Giessen, Germany; anne-christine.aust@chiru.med.uni-giessen.de (A.C.A.); undraga.schagdarsurengin@chiru.med.uni-giessen.de (U.S.); florian.wagenlehner@chiru.med.uni-giessen.de (F.W.); 2 Institute of Organic Chemistry, Justus Liebig University, Heinrich-Buff-Ring 17, 35392 Giessen, Germany; raffael.wende@org.chemie.uni-giessen.de; 3 Molecular Andrology and Urology, Justus Liebig University, Schubertstrasse 81, 35392 Giessen, Germany

**Keywords:** n-undecyl-α/β-l-fucopyranoside, antimicrobial, biofilm, catheter, curcumin, 1-monolaurin, terrein, *P. aeruginosa*, urinary tract infection

## Abstract

**Background/Objectives**: Emerging biofilms of uropathogenic bacteria, particularly *P. aeruginosa*, on medical devices such as urinary catheters, lead to complications in the treatment of urinary tract infections (UTI). Considering the spread of antibiotic resistance, the search for alternative efficient control options for biofilms is of great medical interest. **Methods**: Curcumin, 1-monolaurin, n-undecyl-α/β-l-fucopyranoside, and the fungal metabolite terrein were investigated for their influence on biofilm formation by *P. aeruginosa* on latex catheter pieces in artificial urine (AU), monitoring the number of colony-forming units per cm Latex-Catheter (CFU/cm Latex-Catheter). **Results**: Significant inhibition of *P. aeruginosa* biofilm formation [55.6% CFU reduction/cm^2^] was observed with the fungal metabolite terrein at 256 µg/mL AU. At a concentration of 512 µg/mL AU, terrein achieved almost complete inhibition of biofilm formation. n-undecyl-α/β-l-fucopyranoside inhibited biofilm formation [58.3% CFU reduction/cm^2^] by *P. aeruginosa* ATCC 27853 at 512 µg/mL AU. Compared to that, it caused an increase in biofilm formation [87.0% CFU increase/cm^2^] by *P. aeruginosa* PA 01 at 256 µg/mL AU. This study is limited by the fact that no investigations into the possible cytotoxicity of the two active substances, terrein and n-undecyl-α/β-l-fucopyranoside, on healthy eukaryotic cells have been carried out. **Conclusions**: Natural substances may be a promising approach to prevent the formation of *P. aeruginosa* biofilms. For antibacterial applications, fungal metabolites, such as terrein, offer a novel approach to prevent biofilms in urological practice.

## 1. Introduction

The biofilms of *P. aeruginosa* pose serious problems in everyday urological practice. 2 million people every year are affected worldwide, of which 90 thousand die from *P. aeruginosa* infection [1]. In this work, *P. aeruginosa* Strain ATCC 27853 serves as a test organism, which forms tenacious biofilms [2]. Biofilms are difficult to treat properly, including in the context of urinary tract infections. Urinary catheters cause approximately 75% nosocomial urinary tract infections [3,4,5]. Biofilm formation is a fast and complex process triggered by the nature of the surface, the interaction of individual bacterial cells with each other, Quorum Sensing [3,5,6], and receptor molecules on biological surfaces [7,8]. Within seconds, *P. aeruginosa* can increase the concentration of the second messenger c-di-GMP [9]. Once the biofilm is established, neither the body’s defences nor antibiotic treatment can eradicate bacteria, regardless of the susceptibility of biofilm-producing *P. aeruginosa* to a certain antibiotic [10,11]. The *P. aeruginosa* strain PA 01 reacts to the presence of aminoglycosides [12] in LB medium with increased formation of alginate, an exopolysaccharide or with increased biofilm formation. Micelles and liposomes stabilized with amphiphilic polymer components and loaded with antibiotics are already used in medicine. One stabilizing polymer with good biocompatibility used for stabilization of micelles or liposomes is Soluplus^®^, a polyvinyl caprolactam–polyvinyl acetate–polyethylene glycol graft copolymer [13]. The high dosing of classical antibiotics needed to treat biofilms can cause severe adverse events. For example, fluoroquinolone antibiotics can cause tendon ruptures [14], and the application of aminoglycoside antibiotics carries the risk of lifelong hearing loss [15]. Another issue is the emergence of antimicrobial resistance against antibiotics [16,17,18]. Due to this issue, plant-based agents, such as essential oils [19] or polyphenols, for example, curcumin [20], are investigated. Ding, T et al. observed the inhibiting and biofilm-mitigating effects of curcumin on Gram-negatives, including *Pseudomonas* spp. [21]. Moreover, the Food and Drug Administration (FDA) recognizes curcumin as safe [22]. Under light irradiation with a wavelength of 405 nm, curcumin showed an inhibition of biofilm formation by *P. aeruginosa*. A lower but still inhibiting effect without light irradiation was also observed [23]. 1-Monolaurin (glycerol-α-monolaurate) shows antibacterial effects, but only against Gram-positive bacteria [24,25]. We considered that detergents could shuttle 1-monolaurin through the outer membrane of the Gram-negative *P. aeruginosa* ATCC 27853. Due to the poor solubility of curcumin and 1-monolaurin, these were investigated together with Soluplus^®^ and, in the case of 1-monolaurin, additionally together with Prontosan^®^ (polyhexanide) for their inhibitory effect against biofilm formation by *P. aeruginosa*. Monovalent low molecular weight derivatives of l-fucose interact with LecB of *P. aeruginosa* and are possible inhibitors for biofilm formation by *P. aeruginosa* [26,27]. Therefore, we have synthesized n-undecyl-α/β-l-fucopyranoside according to the modified Fischer synthesis [28] and tested it as an inhibitor of biofilm formation by *P. aeruginosa*. The antibacterial agent terrein (4,5-dihydroxy-3-(1-propen-1-yl)-2-cyclopenten-1-one) from *Aspergillus terreus* acts effectively against the development of *P. aeruginosa* biofilms. It antagonizes quorum sensing of *P. aeruginosa,* acting as a dual inhibitor of QS and c-di-GMP signalling [29]. However, terrein does not affect *P. aeruginosa* cell growth [29]. The fungal metabolite terrein showed efficacy against biofilm formation by *P. aeruginosa* alone and in the presence of the detergent Prontosan^®^. The objective of this study is to evaluate effective alternatives to antibiotics for combating biofilm growth, especially on urological medical devices such as catheters.

## 2. Results

### 2.1. Chemical Synthesis of n-Undecyl-α/β-l-Fucopyranoside

The yield for the in-house synthetized n-undecyl-α/β-l-fucopyranoside was 2.297 g (7.213 mmol; 60%) related to the 1.96 g (11.95 mmol) of α/β-l-fucose used, and the α/β-ratio was ≈ 2:1, according to ^1^H NMR analysis (Supplementary Materials Figure S1).

### 2.2. MIC Values

The MIC values are shown in Table 1. For Piperacillin, the MIC value of 2–4 µg/mL in iron-depleted Mueller–Hinton broth, according to EUCAST standards, was confirmed with our result of 4 µg/mL AU as MIC for *P. aeruginosa* ATCC 27853. In combination with Soluplus^®^, the MIC for Piperacillin did not change. Surprisingly, the detergent Soluplus^®^ did not show any antibacterial effect. Prontosan^®^ showed an inhibitory effect against *P. aeruginosa* cells. The determined MIC value of 16/32 µg/mL AU corresponds to the value that is reported in the literature. For the MIC value of terrein, no comparable value in the literature was found. The combination of terrein and Prontosan^®^ resulted in a reduction in the MIC for terrein. 1-Monolaurin showed no antibacterial effect. The combination with Soluplus^®^ also did not cause any bacteriostatic effect of 1-monolaurin. We observed the same when combining curcumin with Soluplus^®^.

### 2.3. Dependence of Biofilm Formation on the Culture Medium

**Figure 1:**
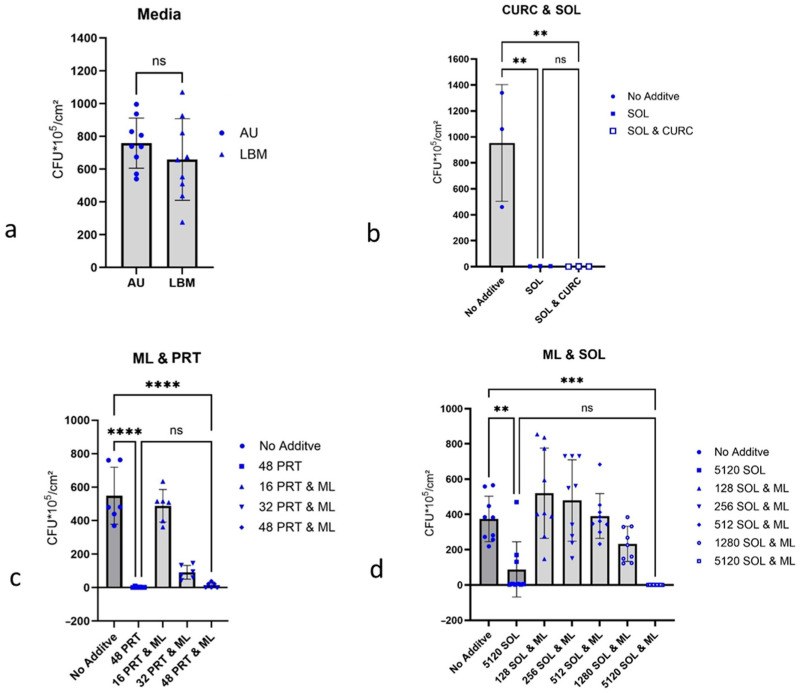
Influence of culture media and low molecular substances on the biofilm growth of *P. aeruginosa* ATCC 27853. Biofilms were determined via counting CFUs; 1 cm latex catheter pieces served as substrate for biofilm growth. The culture medium for tests was AU, and the incubation time was 24 h at 37 °C. (**a**) Comparing two different media; left: artificial urine (AU), right: Lysogeny Broth Medium (LBM). For statistical analysis a two-tailed *t*-test was used instead of an ANOVA analysis. (**b**) 512 µg/mL curcumin (CURC) and 5120 µg/mL Soluplus^®^ (SOL), (**c**) 256 µg/mL 1-monolaurin (ML) and 48 µg/mL Prontosan^®^ (PRT), and (**d**) 512 µg/mL 1-monolaurin (ML) and 5120 µg/mL Soluplus^®^ (SOL). ** for *p* ≤ 0.01; *** *p* ≤ 0.001 and **** *p* < 0.0001 indicates the degree of significance.; ns indicates no significance.

The primary culture of *P. aeruginosa* ATCC 27853, cultivated in AU and LBM, formed biofilms in both cases. The colony numbers from these biofilms grown in AU or LBM showed no significant difference (Figure 1a).

### 2.4. Growth Inhibition of P. aeruginosa Biofilms

#### 2.4.1. Curcumin and 1-Monolaurin, Each with Detergent

Soluplus^®^ alone already showed a strong inhibitory effect, and in combination with curcumin, it masked the possible efficacy of curcumin (Figure 1b). Therefore, further tests with curcumin have not been carried out. 1-Monolaurin showed, in combination with Prontosan^®^, no additional effect (Figure 1c), with Soluplus^®^ additive inhibition against biofilm formation by *P. aeruginosa* ATCC 27853 proving to be insignificant (Figure 1d).

#### 2.4.2. n-Undecyl-α/β-l-Fucopyranoside

**Figure 2:**
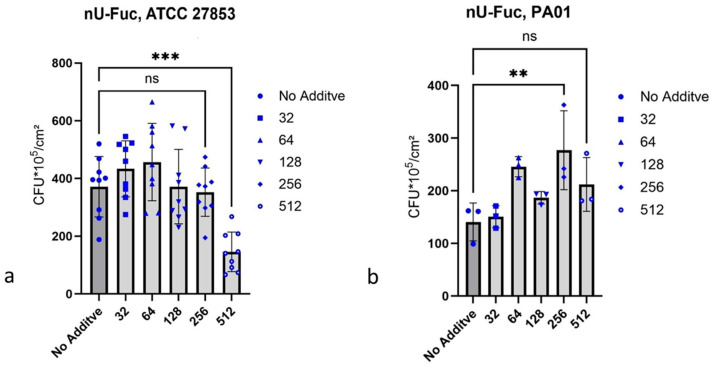
n-Undecyl-α/β-l-fucopyranoside, an inhibitor of the biofilm growth of *P. aeruginosa* ATCC 27853. Biofilms were determined via counting CFUs; 1 cm latex catheter pieces served as the substrate for biofilm growth. The culture medium for tests was AU, and the incubation time was 24 h at 37 °C. (**a**) n-Undecyl-α/β-l-fucopyranoside (nU-Fuc) in different amounts, µg/mL; *** for *p* ≤ 0.001. (**b**) n-Undecyl-α/β-l-fucopyranoside (nU-Fuc) in 32–512 µg/mL as inhibitor of biofilm growth of *P. aeruginosa* PA 01; ** for *p* ≤ 0.01. The *p*-value indicates the degree of significance; ns indicates no significance.

n-Undecyl-α/β-l-fucopyranoside showed 58.3% inhibition of biofilm formation by *P. aeruginosa* ATCC 27853 at a concentration of 512 µg/mL (Figure 2a). In contrast, n-undecyl-α/β-l-fucopyranoside did not inhibit biofilm formation by *P. aeruginosa* PA 01, and at a concentration of 256 µg/mL, it caused an 87.0% increase in biofilm formation. Between n-undecyl-α/β-l-fucopyranoside concentrations of 64 µg/mL to 256 µg/mL, an increase in biofilm formation of 87% occurred (Figure 2b).

#### 2.4.3. Terrein

**Figure 3:**
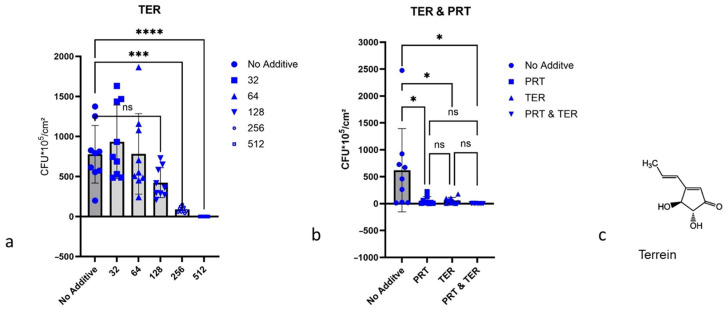
Influence of terrein on the biofilm growth of *P. aeruginosa* ATCC 27853. Biofilms were determined via counting CFUs. 1 cm latex catheter pieces served as the substrate for biofilm growth. The culture medium for tests was AU, and the incubation time was 24 h at 37 °C. (**a**) Terrein (TER) in different concentrations, µg/mL; *** for *p* < 0.001, **** *p* < 0.0001. (**b**) 256 µg/mL terrein and 64 µg/mL Prontosan^®^ (PRT); * for *p* < 0.05. (**c**) Molecular structure of terrein The *p*-value indicates the degree of significance; ns indicates no significance.

Terrein showed 55.6% inhibition of biofilm formation by *P. aeruginosa* ATCC 27853 at a concentration of 256 µg/mL AU, and at 512 µg/mL AU, terrein caused nearly complete inhibition of biofilm formation (Figure 3a). It seems that terrein and the disinfectant Prontosan^®^, tested in combination against *P. aeruginosa* ATCC 27853 biofilm formation, have a synergistic effect against biofilm formation by *P. aeruginosa* ATCC 27853, but significance cannot be confirmed (Figure 3b).

## 3. Discussion

We investigated the impact of curcumin [21], n-undecyl-α/β-l-fucopyranoside [26,27,28], terrein [29,30], and 1-monolaurin [24,25] in terms of the inhibitory effect on the biofilm formation of *P. aeruginosa* ATCC 27853 and *P. aeruginosa* PA 01. The MIC values for the substances tested against *P. aeruginosa* ATCC 27853 showed that, for Soluplus^®^, the antibiofilm and bactericidal effects do not necessarily correlate. Soluplus^®^ neither reduces cell growth nor kills *Pseudomonas* cells; it prevents only the biofilm formation by *P. aeruginosa* ATCC 27853. The influence of the two media, LBM and AU, on the growth of *P. aeruginosa* ATCC 27853 was not significant. Considering the descriptions of Soluplus^®^ [13], we have chosen very high concentrations of Soluplus^®^ for pre-studies with curcumin, circumventing solubility issues with this substance in AU. Dissolving Soluplus^®^ or Prontosan^®^ in AU worked well in the high concentrations chosen. Concentrations of up to 512 µg curcumin in the presence of Soluplus^®^ resulted in stable suspensions. Due to their insolubility in the AU culture medium used, inhibition tests with curcumin or 1-monolaurin alone were not feasible in these preliminary studies. Curcumin, dissolved as a stem solution in a 50 mg/mL concentration in DMSO, precipitated in AU even in a 0.4 mg/mL concentration. However, both detergents overlap with the already proven antibacterial effects of curcumin or possibly of 1-monolaurin on *P. aeruginosa*. Both Soluplus^®^ and Prontosan^®^, at the concentrations applied, caused the near-complete inhibition of biofilm formed by *P. aeruginosa*. In the case of Soluplus^®^, the necessary concentrations used were too high for reasonable usage in medicine. Bioorganic excipients, such as milk powder, for example, may be an alternative to detergents for dissolving curcumin in AU and may facilitate the estimation of a possible inhibitory effect on *P. aeruginosa* biofilm formation on latex catheter surfaces. Unlike the detergents Soluplus^®^ or Prontosan^®^, they do not inhibit biofilm formation. Tested as a possible antilectin, n-undecyl-α/β-l-fucopyranoside led to a significant reduction in biofilm formation by *P. aeruginosa* ATCC 27853, whereas n-undecyl-α/β-l-fucopyranoside was ineffective against *P. aeruginosa* PA 01. The varying expressions of LecB in different *P. aeruginosa* strains could explain this. Due to the negative result obtained, further tests with n-undecyl-α/β-l-fucopyranoside against *P. aeruginosa* PA 01 were not performed. This result may be due to the high genomic diversity of *P. aeruginosa* among different isolates [31]. Terrein was considered nontoxic [30], and, therefore, it is a candidate for potential therapeutic use. Terrein showed a significant inhibitory effect against biofilm formation by *P. aeruginosa* ATCC 27853. Prontosan^®^ did not significantly increase the effect of terrein. The promising effects of terrein, observed by us as a novel inhibitor of biofilm formation, must be tested on further *P. aeruginosa* strains and on other uropathogenic bacteria. Furthermore, the cytotoxicity of terrein and n-undecyl-α/β-l-fucopyranoside on urogenital tract epithelial cells must be tested before medical use against biofilm-associated *P. aeruginosa* infections in urological practice. Due to the interference of terrein with breast cancer cells, toxicity studies with bladder epithelial cells are necessary to assess possible adverse effects on healthy eukaryotic cells [32]. To our knowledge, terrein and the easily accessible n-undecyl-α/β-l-fucopyranoside have not yet been tested for their potential to inhibit biofilm formation on latex catheter surfaces. Our focus of interest was to reveal the number of viable cells within a biofilm, so we did not apply any further methodology than CFU estimation.

## 4. Materials and Methods

### 4.1. Substances and Devices

#### 4.1.1. Culture Media

Artificial urine (AU), as a medium for *P. aeruginosa* test cultures, was freshly prepared according to the recipe mentioned in the literature [19,33]. AU was sterilized by filtration through a 0.2 µm capsule filter (Thermo Fisher Scientific (Waltham, MA, USA) # Cat-No: 10526952) before the addition of autoclaved Tryptone Soy Broth (TSB, Oxoid Ltd., Basingstoke, UK) as a nutritional supplement. The other media used for the cultivation, storage, and testing of *P. aeruginosa* were glycerol ≥99% (Merck Millipore (Burlington, MA, USA) # CAS 56-81-5), Lysogeny Broth Medium (LBM) (Serva Electrophoresis GmbH (Heidelberg, Germany) # Cat-No. 48501.01), and Tryptone Soy Broth (TSB) (Oxoid # Code: CMO129B).

#### 4.1.2. Chemicals and Supporting Material

Dulbecco’s phosphate-buffered saline (PBS) was purchased from Capricorn Scientific GmbH (Ebsdorfergrund, Germany, # Cat-No: PBS-1A). For chemical syntheses and their monitoring, α/β-l-fucose (Carl Roth # CAS No. 2438-80-4), pH indicator rod pH-Fix 0–14 (Carl Roth # Art. No. 0549.2), para-n-dodecyl benzene sulfonic acid (Sigma-Aldrich (St. Louis, MO, USA) # CAS No. 121-65-3), ethyl acetate (Sigma-Aldrich # CAS-No. 141-78-6), n-undecanol (Sigma-Aldrich # CAS-No. 112-42-5), ethanol ≥99,8% (Merck Millipore # CAS 64-17-5), phosphomolybdic acid monohydrate (Merck Millipore # CAS 51429-74-4), sodium hydroxide (Merck Millipore # CAS-No.: 1310-73-2), silica gel 60 (Merck Millipore # CAS No. 7631-86-9), and TLC silica gel 60 Aluminum Sheets 5 × 10 cm (Merck Millipore # Cat-No. 1.16835.0001) were used. Piperacillin (Sigma-Aldrich # CAS-No. 66258-76-2) served as a comparison to assess efficacy. As biofilm-inhibiting substances, curcumin (Merck Millipore # CAS 458-37-7; ≥80% purity), and 1-monolaurin (TCI (Tokyo, Japan) # CAS RN^®^: 142-18-7; >98% purity) were used. The other substances tested as inhibitors were Soluplus^®^ (BASF SE, Ludwigshafen am Rhein, Germany), Prontosan^®^ (B. Braun Melsungen AG, Melsungen, Germany), and terrein (AdipoGen # CAS-No. 582-46-7; ≥98% purity). For terrein and n-undecyl-α/β-l-fucopyranoside, synthesized by us, dimethyl sulfoxide (DMSO) (Sigma-Aldrich # CAS-No. 67-68-5) served as solvent. The substances tested as inhibitors of biofilm formation are shown in Table 2.

#### 4.1.3. Devices

For incubating the bacterial cultures, an IncuLine IL 112 Prime.390-0910 (VWR International GmbH, Darmstadt, Germany) and a Shaking Incubator IKA^®^ KS 4000 i Control (IKA-Werke GmbH and Co. KG, Staufen, Germany) were used. To monitor the bacterial growth, a Laxco MicroSpek™ DSM-Series Cell Density Metre (LAXCO Bothell, Mill Creek, WA, USA) and semi-micro Cuvettes Polystyrene 45 × 12 mm (Sarstedt AG & Co.KG, Nümbrecht, Germany) were used. For catheter testing, Rüsch Gold Latex Catheters (Teleflex Medical GmbH, Fellbach, Germany) served as a substrate for biofilm growth. An ultrasonic device Bandelin SONOREX TK30 50 kHz (BANDELIN electronic GmbH & Co. KG, Berlin, Germany) and a Vortex Scientific Industries Vortex Genie 2 G560 S/N 2-417489 (Thermo Fisher Scientific GmbH, Langerwehe, Germany) were used to process the biofilms and subsequently determine the CFU count (see 4.7). The determination of the minimal inhibitory concentration (MIC) values was performed with 96-well plates checkerboard TC-Plate 96 Well flat-bottom Standard (Sarstedt AG & Co.KG, Nümbrecht, Germany) and a MultiScanGO (Thermo Fisher Scientific GmbH, Langerwehe, Germany).

### 4.2. Organisms

*P. aeruginosa* ATCC 27853 was obtained from ATCC (American Type Culture Collection; Manassas, VA, USA), and *P. aeruginosa* PA 01 from DSMZ (German Collection of Microorganisms and Cell Cultures; Braunschweig, Niedersachsen, Germany).

### 4.3. Culture Conditions

#### 4.3.1. Primary Culture

The cultivation of *P. aeruginosa* and the biofilm inhibition studies on it were performed as follows: From the glycerol stock stored at −80 °C, an inoculum was streaked onto an LB agar plate using a sterile inoculating loop, according to the three-line method, and incubated for 16 h at 37 °C. For the culture in sterile liquid medium (LB medium or AU), 5 mL of medium was placed in a 100 mL Erlenmeyer flask. A single colony was then transferred from the fresh LB agar culture to the medium using a sterile inoculating loop. The culture mixture was then incubated overnight in a shaking incubator at +37 °C and 180 rpm (revolutions per minute).

#### 4.3.2. Secondary Culture

Three 1 cm long catheter pieces were placed as three technical replicates in a sterilized 100 mL Erlenmeyer flask containing 8 mL of LB medium. The test substance was then added to the LB medium at the appropriate concentration. Finally, 100 µL of the *P. aeruginosa* ATCC 27853 primary culture, diluted with sterile PBS buffer to an optical density (OD_λ=600 nm_) of 0.02, was added. After incubation for 24 h at 37 °C, the catheter pieces were removed from the culture medium and were ready for processing (see Section 4.7).

### 4.4. Chemical Synthesis of n-Undecyl-α/β-l-Fucopyranoside

Firstly, 1.96 g α/β-l-fucose (11.95 mmol), 24.69 mL n-undecanol (20.49 g = 118.90 mmol), 1.832 mL (1.942 g = 5.98 mmol) para-n-dodecylbenzenesulfonic acid, and 190 μL fully desalinated H_2_O were stirred in a closed 25 mL Schott flask at 60 °C and 700 rpm for 24 h. The initial yellowish-white suspension was a slightly cloudy mixture after the reaction ended. To monitor the reaction process, thin layer chromatography was performed (eluent: ethyl acetate/ethanol 90:10). Unreacted n-undecanol was removed via column chromatography on silica gel eluting with ethyl acetate/ethanol (100:0 then 90:10) [28]. The product was a white crystalline solid. The yield was 60%, related to the α/β-l-fucose used; see 2.1. The identity of the product was confirmed by mass spectrometry (MW = 318.35 g) and nuclear magnetic resonance spectroscopy (NMR). HRMS (ESI): *m*/*z* calculated for C_17_H_34_O_5_Na [M + Na]^+^: 341.2299; found: 341.2297. ^1^H NMR (600 MHz, CD_3_OD): δ 4.73 (d, *J* = 2.7 Hz, 1 H, H-1α), 68% for n-undecyl-α-l-fucopyranoside, 4.17 (d, *J* = 7.5 Hz, 0.5 H, H-1β), 32% for n-undecyl-β-l-fucopyranoside, 3.97–3.91 (m, 1 H, H-5α), 3.83 (dt, *J* = 9.5, 6.8 Hz, 0.5 H, β–O*CH_2_CH*_2_), 3.74–3.70 (m, 2 H, H-2α/H-3α), 3.68–3.58 (m, 3 H, α–OC*H*_2_CH_2_/H-4α/H-6β), 3.51 (dt, *J* = 9.5, 6.7 Hz, 0.5 H, β–OC*H*_2_CH_2_), 3.47–3.41 (m, 2 H, α–OC*H*_2_CH_2_, H-4β, H-4β), 1.72–1.50 (m, 3 H, α–OCH_2_C*H*_2_/β–OCH_2_C*H*_2_), 1.43–1.23 (m, 25.5 H, α-C*H*_2_/β-C*H*_2_/H-6β), 1.21 (d, *J* = 6.6 Hz, 3 H, H-6α), 0.90 (t, *J* = 6.9 Hz, 4.5 H, α-C*H*_3_/β-C*H*_3_) ppm. ^13^C{^1^H} NMR (150 MHz, CD_3_OD): δ 104.8 (C-1β), 100.5 (C-1α), 75.2 (C-5β), 73.6 (C-4α), 73.1 (C-3β), 72.3 (C-2β), 71.8 (C-4β), 71.7 (C-3α), 70.8 (β–O*C*H_2_), 70.0 (C-2α), 69.3 (α–O*C*H_2_), 67.5 (C-5α), 33.1, 30.9, 30.8, 30.7, 30.6, 30.6, 30.6, 30.5, 27.3, 27.1, 23.7 (α-*C*H_2_/β-*C*H_2_), 16.8 (C-6β), 16.7 (C-6α), 14.4 (α-*C*H_3_/β-*C*H_3_) ppm. δ [ppm] indicates the chemical shift in an atomic nucleus relative to the solvent residual peak in a magnetic field, *J* [Hz] means the coupling constant between neighbouring atomic nuclei, and multiplet abbreviations mean the signal multiplicity. This indicates the number of adjacent atomic nuclei connected to neighbouring nuclei. More information can be found in the relevant textbooks on nuclear magnetic resonance spectroscopy [36].

### 4.5. Determination of Minimal Inhibitory Concentration

The MIC values were determined according to EUCAST standards [33,35] for antibacterial agents by broth dilution. From the agent to be tested, a two-fold dilution series with a volume of 160 µL per well was made in AU. Then, every well containing agent was inoculated with 10 µL of PBS buffer-diluted *P. aeruginosa* ATCC 27853 suspension (1:200 *v*/*v*). The agents tested, listed in alphabetical order with highest concentration in µg/mL [number shown in parentheses] were curcumin and Soluplus^®^ [512 _(curcumin)_ and 5120 _(Soluplus_^®^_)_], 1-Monolaurin and Soluplus^®^ [512 _(1-Monolaurin)_ and 5120 _(Soluplus_^®^_)_], Prontosan^®^ [512], Soluplus^®^ [5120], terrein [1024], and terrein and Prontosan^®^ [1024 _(terrein)_ and 32 _(Prontosan_^®^_)_]. The concentration of the detergents Soluplus^®^ and Prontosan^®^ we held constant throughout the dilution series. Since we expected no inhibiting effect for n-undecyl-α/β-l-fucopyranoside, and curcumin, which is insoluble in aqueous media, no MICs were determined for these two substances. The medium without agent inoculation served as a positive control, and the medium without bacteria and agent served as a negative control. After overnight incubation at 37 °C, turbidity was measured at λ = 600 nm. MIC means the lowest concentration at which no additional turbidity (corresponding to bacterial growth) can be detected compared to the positive control. The MIC values shown are the median values calculated from three biological replicates. Each biological replicate consisted of three dilution series on 96-well plates as technical replicates, whose results were averaged. Piperacillin served as positive standard; we used it in every MIC test we performed (Table 1).

### 4.6. Catheter Biofilm Experiments

#### 4.6.1. General Procedure Catheter Experiment

**Figure 4:**
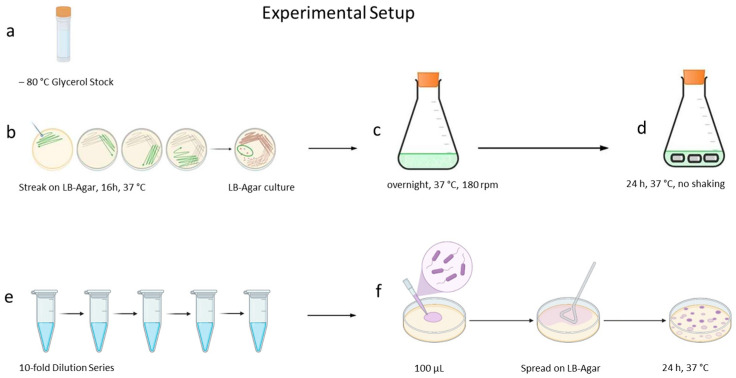
Experimental setup. (**a**) −80 °C glycerol stock of *P. aeruginosa* ATCC 27853, (**b**) the spread on LB agar with a following 16 h incubation, (**c**) primary culture of *P. aeruginosa* ATCC 27853, (**d**) secondary culture with three catheter pieces in medium, (**e**) ten-fold dilution series made from biofilm suspension obtained from processed catheter-pieces, and (**f**) the spread appropriate dilutions on LB agar with following 24 h incubation for CFU determination.

To 8 mL of medium, three 1 cm long catheter segments, as technical replicates, and the substance to be tested, 100 µL of a freshly cultivated (Figure 4a–c) PBS-buffer-diluted *P. aeruginosa* ATCC 27853 suspension, was added. Incubation for 24 h to 36 h at 37 °C followed, and, after processing, the grown biofilms, CFUs, were counted (see Section 4.7). Each test series was biologically replicated three times (Figure 4c–f).

#### 4.6.2. Influence of the Culture Medium

Due to the results shown for *Proteus mirabilis* [37], differences in the growth behaviour of *P. aeruginosa* ATCC 27853 in AU and LB medium were estimated. To 8 mL of AU and LB medium without additives and to three 1 cm long latex catheter segments, respectively, *P. aeruginosa* ATCC 27853 suspension with an OD_λ=600nm_ = 0.02 in PBS buffer was added. After incubation for 24 h at 37 °C, the colonies were counted (Figure 4c–f).

#### 4.6.3. Curcumin and 1-Monolaurin, Each in Combination with Soluplus^®^

To 8 mL of AU and three 1 cm long catheter segments, 512 µg/mL curcumin together with 5120 µg/mL Soluplus^®^ were added and incubated over 24 h at 37 °C in the presence of *P. aeruginosa* ATCC 27853 according to the general procedure. Three 1 cm long catheter segments and *P. aeruginosa* ATCC 27853 without an additive in AU and with 5120 µg/mL Soluplus^®^ without further additives served as control. In the same manner, 512 µg/mL AU 1-monolaurin together with 48 µg/mL AU Prontosan^®^ and 128 µg to 5120 µg/mL AU Soluplus^®^ were tested (Figure 4).

#### 4.6.4. n-Undecyl-α/β-l-Fucopyranoside

n-Undecyl-α/β-l-fucopyranoside was tested in concentrations from 32 µg/mL AU to 512 µg/mL AU following the general procedure, against biofilm formation by *P. aeruginosa* ATCC 27853 and by *P. aeruginosa* PA 01 (Figure 4).

#### 4.6.5. Terrein

Terrein, an antimicrobial metabolite from the mould *A. terreus*, was tested at concentrations from 32 µg/mL AU to 512 µg/mL AU following the general procedure (Figure 4). To estimate possible co-effects of terrein and Prontosan^®^, 256 µg/mL AU terrein together with 64 µg/mL AU Prontosan^®^ were tested for the mitigation of biofilm formation by *P. aeruginosa* ATCC 27853. Because keratinocytes tolerate Prontosan^®^ well in concentrations up to 175 µg/mL [34], we have chosen Prontosan^®^ as a co-agent instead of Soluplus^®^.

### 4.7. Estimation of Colony-Forming Units in the Biofilm

The biofilm processing procedure is as follows: After incubation, the 1 cm catheter pieces were transferred from the Erlenmeyer flask into an Eppendorf tube containing 1000 µL of PBS buffer using sterile tweezers and gently swirled. This was repeated two more times with 1000 µL of PBS buffer to ensure that all free-floating cells were washed out of the biofilm. To suspend the living cells within the biofilm, the catheter piece was sonicated at 50 kHz in 1000 µL of PBS buffer for 5 min, vortexed for 2 min, sonicated again for 5 min, and vortexed for 1 min. A ten-fold dilution series was prepared from the resulting biofilm suspension. Then, 100 µL of the suitable dilutions (10^−4^ to 10^−7^) were spread onto the LB agar plates. After incubating the LB agar plates at 37 °C for 16 h, the colonies were counted [34,38].

### 4.8. Statistical Analysis

For statistical analyses, we used GraphPad Prism 10; version: GraphPad Prism 10.4.2. Statistical significance was set as *p* ≤ 0.05, with α = 0.05. The degree of significance is shown using * for *p* ≤ 0.05, ** *p* ≤ 0.01, *** *p* ≤ 0.001, and **** *p* ≤ 0.0001. For the statistical comparison of two data sets with GraphPad Prism 10.4.2 a two-tailed *t*-test was used. The one-way ANOVA test served for the analysis of more than three data sets. The ANOVA–Dunnett test served to compare multiple data sets with a single control. The Tukey test was used to compare every data set with every other.

## 5. Conclusions

The two active ingredients, n-undecyl-α/β-l-fucopyranoside and terrein, tested for the first time on latex catheters for urological practice, show an inhibition of biofilm formation by *P. aeruginosa* ATCC 27853. The chemically pure anomers of n-undecyl-α/β-l-fucopyranoside are necessary to specify their efficacy as inhibitors of biofilm formation by *P. aeruginosa*. Another goal for the future is to estimate the mitigating effects of terrein on established biofilms and test the potential of terrein to prevent the formation of biofilms by other bacterial strains.

## Figures and Tables

**Table 1:** MIC values [µg/mL] in AU *P. aeruginosa* ATCC 27853.

Substance	MIC [µg/mL]
Curcumin and Soluplus^®^	>512 _(Curcumin)_ and >5120 _(Soluplus_^®^_)_
Curcumin	not determined
Soluplus^®^	>5120
1-Monolaurin	>512
1-Monolaurin and Soluplus^®^	>512 _(1-Monolaurin)_ and >5120 _(Soluplus_^®^_)_
Prontosan^®^	16/32
Terrein	512
Terrein and Prontosan^®^	128 _(Terrein)_ and 32 _(Prontosan_^®^_)_
n-Undecyl-α/β-l-Fucopyranoside	not determined
Piperacillin	4

Note: The minimal inhibitory concentration (MIC) value of n-undecyl-α/β-l-fucopyranoside is not determined in AU (artificial urine).

**Table 2:** Overview of substances tested for inhibition of biofilm formation by *P. aeruginosa*.

Substance	Abbreviation	Literature	Vendor
Curcumin and Soluplus^®^	CURC and SOL		
Curcumin	CURC	Abdulrahman et al. 2020 [23]	Merck Millipore # CAS 458-37-7
Soluplus^®^	SOL	Hendrik Hardung et al. [13]	BASF SE Ludwigshafen am Rhein Germany
1-Monolaurin	ML	Oh, and Marshall 1993 [24]; Kabara et al. 1972 [25]	TCI # CAS RN^®^: 142-18-7
1-Monolaurin & Soluplus^®^	ML and SOL		
Prontosan^®^	PRT	Loose et al. 2021 [34]	B. Braun Melsungen AG, Melsungen, Germany
Terrein	TER	Kim et al. 2018 [29]	AdipoGen # CAS-No. 582-46-7
Terrein and Prontosan^®^	TER and PRT		
n-Undecyl-α/β-l-Fucopyranoside	nU-Fuc	Nowicki et al. 2017 [28]; Sommer et al. 2018 [27]	Laboratory synthesis
Piperacillin	PIP	EUCAST QC Tables [35]	Sigma-Aldrich # CAS-No. 66258-76-2

## Data Availability

All data generated and analyzed during this work are included in this published manuscript.

## Supplementary Materials

The following supporting information can be downloaded at: https://www.mdpi.com/article/10.3390/antibiotics15010076/s1. Figure S1: Spectral analysis of n-undecyl-α/β-l-fucopyranoside. (**a**) Mass spectrum n-undecyl-α/β-l-fucopyranoside. (**b**) 1H NMR spectrum n-undecyl-α/β-l-fucopyranoside; α/β-ratio: ≈ 2:1. (**c**) 1H NMR spectrum n-undecyl-α/β-l-fucopyranoside; α/β-ratio: ≈ 2:1, zoom. (**d**) 13C NMR spectrum n-undecyl-α/β-l-fucopyranoside; α/β-ratio ≈ 2:1.

## Abbreviations

The following abbreviations are used in this manuscript (in alphabetical order):

ATCC	American Type Cell Collection
AU	Artificial Urine
c-di GMP	Cyclic di-Guanosine Monophosphate
CFU	Colony-Forming Unit
CURC	Curcumin
DMSO	Dimethyl Sulfoxide
DSMZ	Deutsche Sammlung von Mikroorganismen und Zellkulturen (= German Collection of Microorganisms and Cell Cultures)
EUCAST	European Committee on Antimicrobial Susceptibility Testing
HRMS	High-Resolution Mass Spectrometry
Hz	Hertz
LBM	Lysogeny Broth Medium
M+	Molecular Ion Peak
ML	1-Monolaurin
MW	Molecular Weight
NMR	Nuclear Magnetic Resonance
nU-Fuc	n-Undecyl-α/β-l-Fucopyranoside
PBS	Phosphate-Buffered Saline
PIP	Piperacillin
ppm	Part per Million
rpm	Revolutions per Minute
PRT	Prontosan®
QC	Quality Control
SOL	Soluplus®
TER	Terrein
TSB	Tryptone Soy Broth
