# Supplementary material for: Activity of Natural Substances and n-Undecyl-α/β-l-Fucopyranoside Against the Formation of Pathogenic Biofilms by Pseudomonas aeruginosa
Source: Antibiotics (Basel). 2026 Jan 10;15(1):76. doi: 10.3390/antibiotics15010076 (PMC12837513; doi:10.3390/antibiotics15010076)
Supplement: Supplementary file 1 [file antibiotics-15-00076-s001.zip › antibiotics-4038797-supplementary.pdf]

Article

Activity of Natural Substances and n-Undecyl-α/β-L-Fucopyranoside Against the Formation of Pathogenic Biofilms by *Pseudomonas aeruginosa*

Christian Dietrich Vogel [1,*], Anne Christine Aust [1], Raffael Christoph Wende [2], Undraga Schagdarsurengin [1,3] and Florian Wagenlehner [1]

[1] Department of Urology, Pediatric Urology and Andrology, Justus Liebig University, Rudolf-Buchheim-Strasse 7, 35392 Giessen, Germany; anne-christine.aust@chiru.med.uni-giessen.de (A.C.A.); undraga.schagdarsurengin@chiru.med.uni-giessen.de (U.S.); florian.wagenlehner@chiru.med.uni-giessen.de (F.W.)

[2] Institute of Organic Chemistry, Justus Liebig University, Heinrich-Buff-Ring 17, 35392 Giessen, Germany; raffael.wende@org.chemie.uni-giessen.de

[3] Molecular Andrology and Urology, Justus Liebig University, Schubertstrasse 81, 35392 Giessen, Germany

* Correspondence: christian.vogel@chiru.med.uni-giessen.de

Supplementary Materials

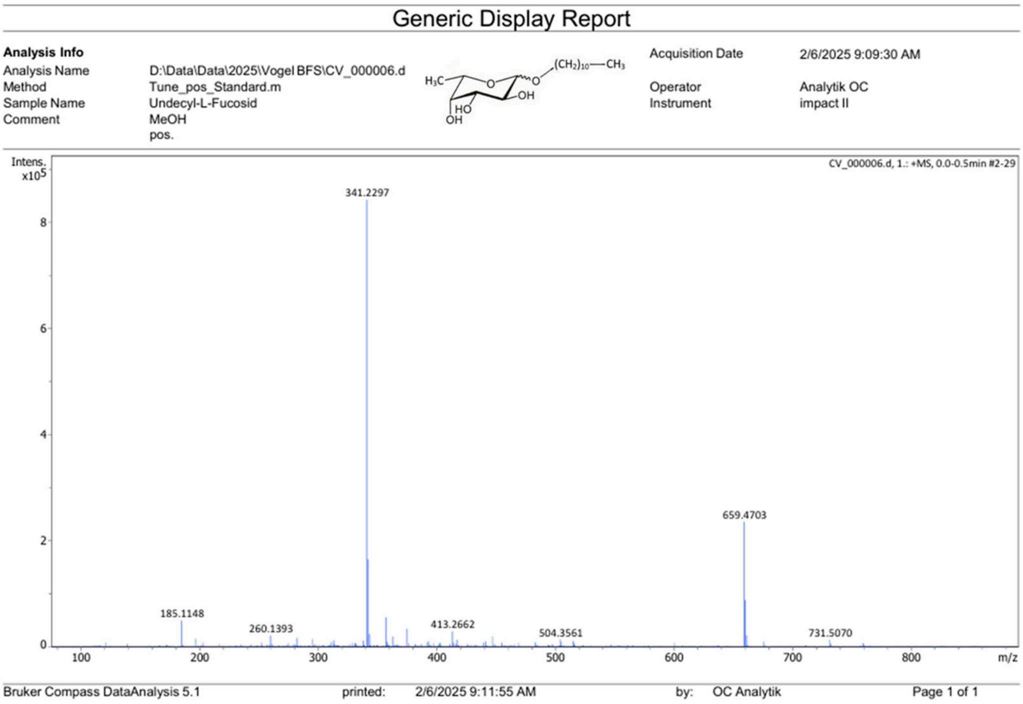

1a) Mass spectrum n-Undecyl-α/β-L-fucopyranoside

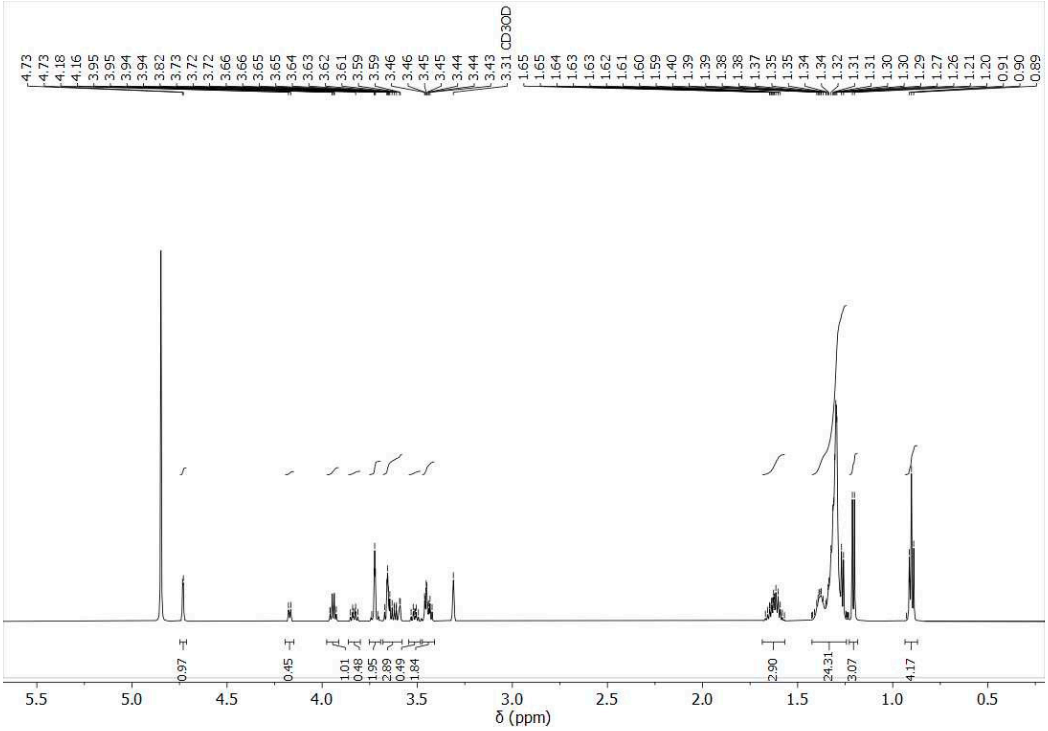

1b) ^{1}H NMR spectrum n-Undecyl-α/β-L-fucopyranoside, α/β-ratio: ≈ 2:1

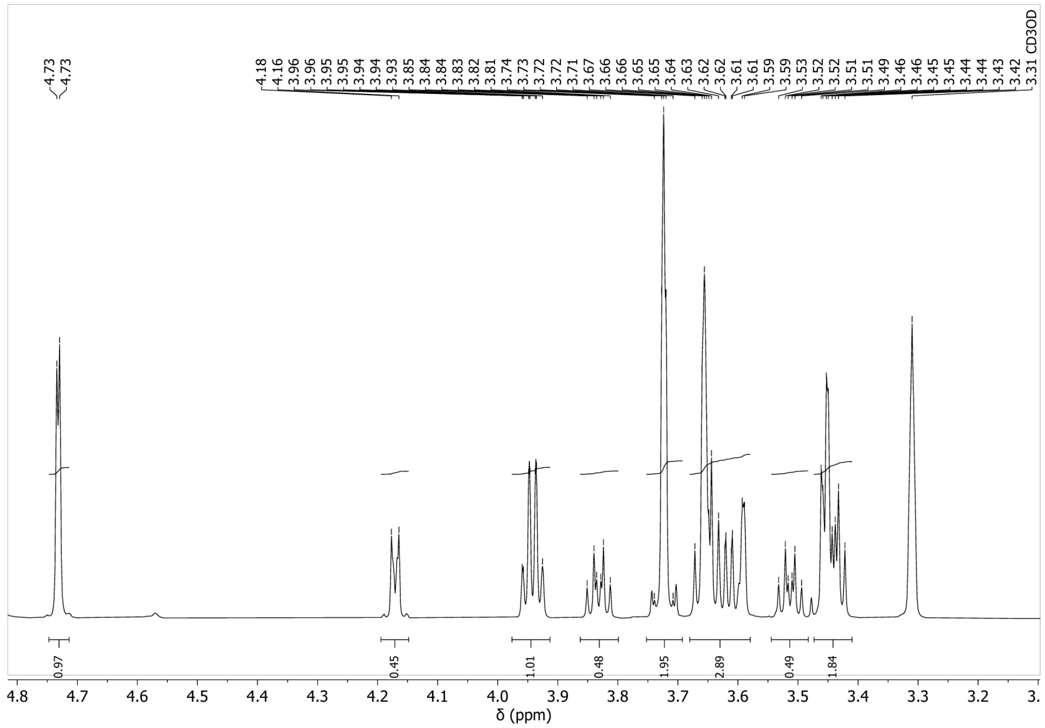

1c) ^{1}H NMR spectrum n-Undecyl-α/β-L-fucopyranoside, α/β-ratio: ≈ 2:1, zoom

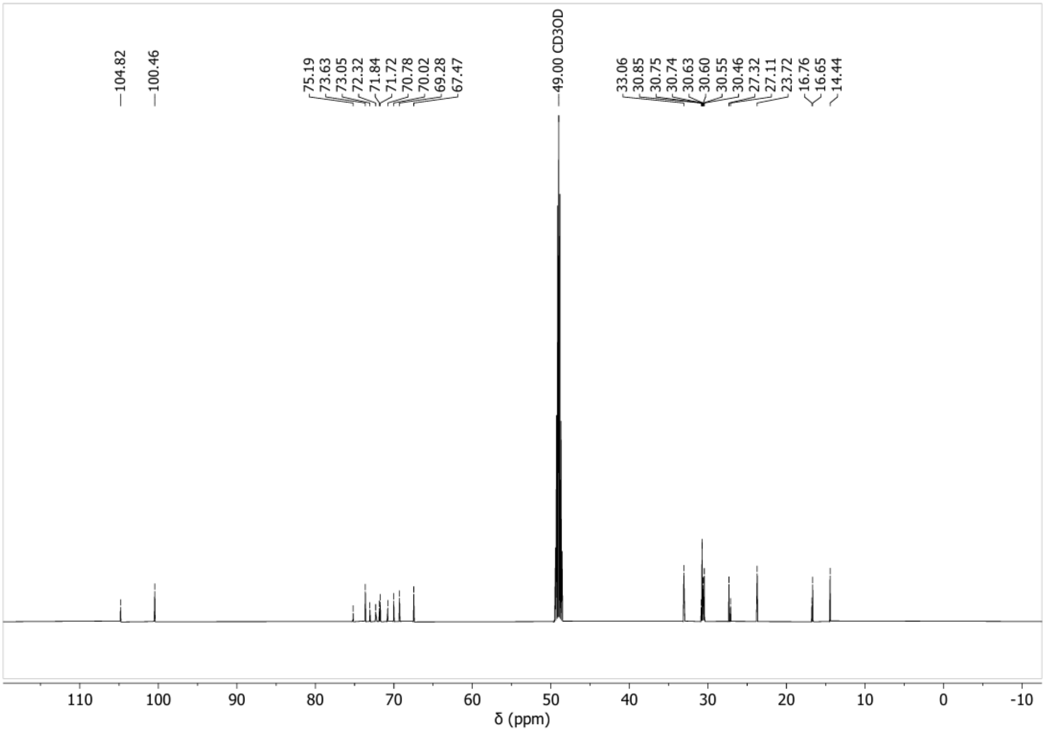

1d) ^{13}C NMR spectrum n- Undecyl-α/β-L-fucopyranoside, α/β-ratio ≈ 2:1

Figure S1. Spectral analysis of n-undecyl-α/β-L-fucopyranoside 1a) Mass spectrum n-Undecyl-α/β-L-fucopyranoside, 1b) ^{1}H NMR spectrum n-Undecyl-α/β-L-fucopyranoside, α/β-ratio: ≈ 2:1, 1c) ^{1}H NMR spectrum n-Undecyl-α/β-L-fucopyranoside, α/β-ratio: ≈ 2:1, zoom, 1d) ^{13}C NMR spectrum n- Undecyl-α/β-L-fucopyranoside, α/β-ratio ≈ 2:1.
